# Extracellular Vesicle‐Based Therapies for Osteoarthritis—Advances, Challenges and Hopes

**DOI:** 10.1002/exp2.70212

**Published:** 2026-08-10

**Authors:** Jiao Jiao Li, Wei Seong Toh

**Affiliations:** ^1^ School of Biomedical Engineering, Faculty of Engineering and IT University of Technology Sydney Sydney New South Wales Australia; ^2^ Kolling Institute, Faculty of Medicine and Health The University of Sydney St Leonards New South Wales Australia; ^3^ Woolcock Institute of Medical Research Macquarie University Sydney New South Wales Australia; ^4^ Department of Orthopaedic Surgery Yong Loo Lin School of Medicine, National University of Singapore Singapore Singapore; ^5^ Faculty of Dentistry National University of Singapore Singapore Singapore; ^6^ NUS Tissue Engineering Programme Life Sciences Institute, National University of Singapore Singapore Singapore; ^7^ Department of Biomedical Engineering College of Design and Engineering, National University of Singapore Singapore Singapore

## Abstract

Recent advances, challenges and hopes in using extracellular vesicles (EVs) as therapeutics for osteoarthritis. Recent advances include native and engineered EVs and translational developments. Challenges include cell sources and targeted effects of EVs, as well as establishing robust critical quality attributes, while hopes for advancing this field arise from emerging technologies in single‐EV analysis, multi‐omics, scalable manufacturing and artificial intelligence (AI).

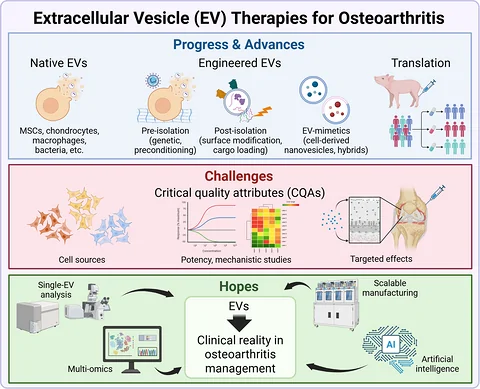

1

Osteoarthritis (OA) is a leading cause of pain, disability and loss of productivity in ageing populations. Existing treatments provide only symptomatic relief, highlighting an urgent need for disease‐modifying OA therapies. In this context, extracellular vesicles (EVs) have emerged as promising *next‐generation* therapeutics. As bioactive cell‐derived membrane particles, EVs can modulate inflammation and reshape the OA microenvironment to promote tissue repair. This commentary examines recent advances in EV‐based therapies for OA, discusses the key challenges and highlights the hopes they hold for redefining the therapeutic landscape.

## 2 Current Progress and Research Advances

**FIGURE 1:**
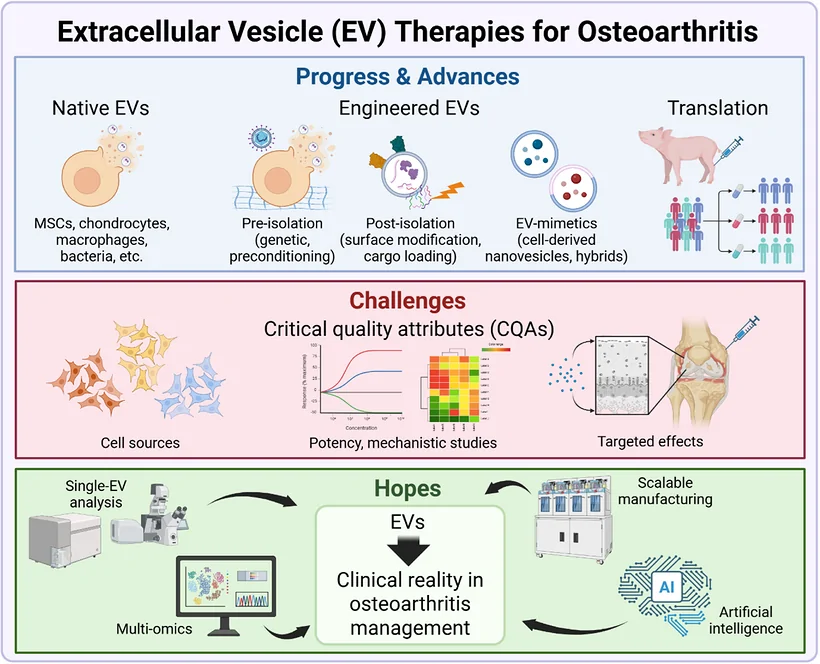
Recent advances, challenges and hopes in using extracellular vesicles (EVs) as therapeutics for osteoarthritis. Image created using BioRender.

Osteoarthritis (OA) affects approximately 600 million people worldwide and is a leading cause of pain, disability, and loss of productivity [1]. As a chronic, degenerative disease that affects the entire joint, OA is characterised by progressive breakdown of articular cartilage, thickening of subchondral bone, degeneration of ligaments (as well as menisci in knee OA), along with varying degrees of synovial inflammation. Although most commonly affecting the knee, OA can also affect the hips, hands, spine, and jaw. Despite its prevalence, OA remains largely refractory to current therapies. Existing pharmacological treatments provide only symptomatic relief, and while cell therapies show promise, their clinical translation is hindered by challenges in maintaining cell viability, potency, and manufacturing consistency. Extracellular vesicles (EVs) have emerged as promising next‐generation therapeutics for OA (Figure 1).

The landmark discovery that mesenchymal stromal cells (MSCs) exert much of their therapeutic activity through paracrine signalling, particularly through EVs, introduced a fundamentally new therapeutic paradigm [2]. Rather than depending on live cells, this approach leverages cell‐derived nanoscale vesicles to deliver complex cargoes of proteins, lipids, nucleic acids and metabolites in a physiologically encoded manner. This insight has catalysed a shift towards ‘cell‐free’ EV therapies, addressing many of the practical limitations of cell‐based treatments while offering greater stability, ease of administration and pharmacological predictability. Early studies in this area demonstrated that EVs released by MSCs can suppress inflammation, stimulate anabolic signalling in chondrocytes and modulate immune cell phenotypes, laying the groundwork for developing EV‐based therapeutics for OA treatment [3, 4]. Since then, a significant volume of research has emerged on MSC‐derived EVs (MSC‐EVs), alongside a growing diversity of non‐MSC sources of EVs, each offering distinct mechanistic insights and promising avenues for OA treatment [5]. Other cell types such as chondrocytes, synovial fibroblasts and macrophages have been shown to secrete EVs capable of mitigating OA in animal models. More recently, EVs from unconventional sources such as milk and bacteria have attracted attention for their ability to modulate systemic pathways, such as the gut–joint axis [6], further expanding the landscape of EV‐based interventions in OA across multiple organ systems and routes of administration. Collectively, this expanding diversity of EV sources underscores the need for rigorous comparative evaluation and rational selection to identify the optimal source for clinical translation.

In parallel with advances in native EV research, engineering strategies are rapidly moving the field towards programmable and more potent or targeted EV‐based therapeutics. Since the composition and therapeutic activity of EVs are closely influenced by the parent cell phenotype, physiological state and surrounding microenvironment, pre‐isolation engineering strategies that modulate these cellular properties have gained increasing popularity. Several strategies have been developed to enhance EV yield and functional properties, including genetic manipulation (e.g., overexpression of specific miRNAs such as miR‐140), biochemical stimulation (e.g., growth factors such as TGF‐β1) and biophysical preconditioning such as 3D culture, hypoxia, hydrostatic pressure and mechanical or electromagnetic field stimulation. These strategies provide effective tools to tune EV production and cargo composition, resulting in EVs enriched with anti‐inflammatory miRNAs and chondroprotective factors, which have enhanced capacity to modulate the inflammatory joint microenvironment and support repair.

Post‐isolation engineering strategies can further enhance the therapeutic potential of native EVs by modifying their surface or loading specific cargo to improve tissue targeting capability and therapeutic efficacy. Surface modification can involve incorporating or attaching molecules such as aptamers, transmembrane receptors, or peptides for directing EVs to specific cell types such as chondrocytes within the joint. For example, umbilical cord‐derived MSC‐EVs modified with a chondrocyte‐affinity peptide (CAP; DWRVIIPPRPSA) selectively targeted OA chondrocytes in vitro and showed superior cartilage‐binding capacity in vivo compared with unmodified EVs [7]. The enhanced cartilage targeting and joint retention capability of these modified EVs facilitated more effective cartilage repair in a rat OA model. Meanwhile, EV cargo loading can be performed through approaches such as electroporation, sonication, freeze‐thaw cycling, or extrusion. Originally developed for introducing exogenous materials into cells, these methods have been adapted to load therapeutic payloads into EVs, although with varying degrees of success. Small molecules (e.g., kartogenin) and nucleic acids (e.g., miRNAs, circRNAs) are examples of factors loaded to enhance the chondroprotective potential of engineered EVs.

To further improve EV loading efficiency, increasing attention has been directed towards the identification and application of molecule sorting modules, which can selectively bind the target cargo and facilitate its incorporation into EVs [8]. This strategy preserves EV membrane integrity while enabling efficient loading of larger protein cargoes. A recent study by Yan et al. [9] identified ectonucleotide pyrophosphatase/phosphodiesterase 1 (ENPP1), specifically a truncated 144‐aa variant (EN144), as a highly effective EV‐sorting protein. Fusion of EN144 to the IL‐6 decoy receptor gp130 enabled efficient incorporation into EVs, producing engineered decoy EVs that potently inhibited IL‐6 trans‐signalling and alleviated tissue damage in OA rats.

In recent years, EV‐mimetic strategies have gained increasing attention, particularly cell‐derived nanovesicles (CDNs). These EV‐mimetics are generated by physically disrupting whole cells, typically through mechanical extrusion or sonication, to produce nanosized vesicles with similar physicochemical characteristics and molecular cargo as EVs, but at substantially higher yields. For example, using bone marrow‐derived MSCs, CDNs were produced at 100‐fold higher yield compared to naturally derived EVs [10]. Importantly, these CDNs exhibited comparable size, morphology and protein‐marker expression and similarly supported chondrocyte proliferation, migration and differentiation, underscoring their potential as a high‐yield alternative to native EVs.

In addition to EV‐mimetics, hybrid EV systems have emerged, which are formed by fusing natural EVs with synthetic nanoparticles such as liposomes or lipid nanoparticles (LNPs). These EV‐LNP hybrids integrate the intrinsic biocompatibility and cell‐targeting properties of EVs with the stability, uniformity and high drug‐loading capacity of synthetic nanocarriers. Such hybrid vesicles can overcome the inherent limitations of natural EVs while enhancing targeted delivery, therapeutic payload and overall efficacy. For example, ethanol‐mediated fusion was employed to create a hybrid system combining TGF‐β1–overexpressed EVs with nicotinamide‐loaded, collagen Type II Alpha 1 (Col2A1) antibody‐modified LNPs [11]. This Ab‐Hybrid maintained EV bioactivity while incorporating the advantages of LNPs, achieving targeted, sustained chondroprotective and anti‐inflammatory effects in a rat OA model, highlighting the therapeutic potential of hybrid EV strategies.

To enhance the bioavailability and therapeutic efficacy of EVs, advanced biomaterial systems have been developed that exploit unique physicochemical properties for EV delivery into musculoskeletal tissues. In these EV‐biomaterial platforms, EVs are immobilised within scaffolds, functionalised with extracellular matrix (ECM)‐binding motifs, or engineered to leverage electrostatic interactions, hence enabling sustained and localised delivery [12]. Decellularised ECM and key ECM components such as collagen, fibronectin and hyaluronic acid (HA) are widely incorporated into these biomaterial platforms to provide structural support while enhancing EV loading, retention and bioactivity [13]. Importantly, EVs carry cargo molecules including fibronectin 1, CD44, integrins and matrix metalloproteinases, which mediate dynamic interactions with surrounding ECM components. These interactions regulate diverse cellular processes and actively drive ECM remodelling during tissue repair. Future research should focus on elucidating the mechanisms that optimise EV‐ECM interactions and guide the rational design of biomaterials for improved EV targeting, sustained release and therapeutic efficacy.

Current progress in EV‐based OA therapy is largely interpreted through demonstrated effects on key pathological features in preclinical models, including cartilage preservation, attenuation of synovial inflammation and modulation of subchondral bone remodelling. However, there is limited evidence on direct, head‐to‐head comparison of therapeutic efficacy among different EV sources or engineering strategies, while substantial variability exists in preclinical OA models (species, induction methods, disease stage and outcome measures). Consequently, although promising mechanistic insights and efficacy signals are emerging, the field lacks standardised frameworks to robustly compare approaches and define optimal EV‐based interventions for OA [5], representing an important direction for future development.

Translational development of EV‐based therapeutics is advancing rapidly, where large animal models are essential for evaluating safety, efficacy and functional mechanical competence in a clinically relevant setting. In a study by Zhang et al. [14], micropigs with osteochondral defects in the medial femoral condyle received three weekly intra‐articular injections of either MSC‐EVs combined with HA or HA alone. After four months, animals treated with MSC‐EVs exhibited substantially improved repair outcomes, evidenced by superior magnetic resonance imaging (MRI) findings and robust regeneration of cartilage and subchondral bone. These findings were further corroborated by significantly improved macroscopic, histological and biomechanical outcomes. Notably, no adverse reactions or systemic alterations were observed, underscoring the safety profile and translational promise of MSC‐EV therapy.

The scientific progress in EV therapeutics parallels strong commercial growth. The global EV market was valued at USD 189.4 million in 2024, increasing to USD 214.4 million in 2025 and is projected to reach USD 480.6 million by 2030 — reflecting a robust compound annual growth rate of 17.5% from 2025 to 2030 [15]. Such rapid expansion reflects escalating interest from industry and investors in EV‐based diagnostics and therapeutics, including those targeting OA treatment.

Globally, clinical translation of EV‐based treatments for cartilage defects and OA is accelerating, with at least five clinical trials currently registered on ClinicalTrials.gov (search terms: ‘extracellular vesicles’, ‘exosomes’, ‘cartilage’, ‘osteoarthritis’, ‘osteoarthrosis’ accessed on April 10, 2026). Table 1 provides an overview of these ongoing clinical efforts. Together, the convergence of promising preclinical data, expanding market investment and growing clinical activity highlights the strong momentum driving EV‐based therapies towards clinical application in cartilage repair and OA management.

**TABLE 1:** Registered clinical trials evaluating EV‐based treatments for cartilage defects and OA (from ClinicalTrials.gov, accessed on April 10, 2026).

NCT number	Title	Condition	Location	Status
NCT05060107	Intra‐articular injection of MSC‐derived Exosomes in knee osteoarthritis (ExoOA‐1)	Knee OA	Unknown	Unknown
NCT06431152	Intra‐articular injection of UC‐MSC exosome in knee osteoarthritis	Knee OA	Santiago, Las Condes, Chile	Recruiting
NCT06466850	Mesenchymal stem cells derived exosomes in osteoarthritis patients	Knee OA	Isfahan, Iran	Recruiting
NCT06713902	Extracellular vesicles in fibrin gel for cartilage repair	Joint Disease	Milan, MI, Italy	Recruiting
NCT06937528	Use of extracellular vesicles (EV) for knee osteoarthrosis	Knee OA	Amman, Jordan	Recruiting

## 3 The Challenges

Despite rapidly growing interest in EV‐based therapeutics for OA, several challenges need to be addressed to accelerate their translation to the clinic. A major hurdle lies in defining the critical quality attributes (CQAs) that ensure consistent product identity and potency, given the variability arising from cell sources, culture conditions, EV enrichment methods and inherent heterogeneity of both producing cells and their EVs.

In the context of OA treatment, primary cells such as MSCs, chondrocytes and synovial fibroblasts hold therapeutic promise as EV‐producing sources, but they also introduce considerable manufacturing challenges. Variability stemming from tissue origin, donor‐to‐donor and intra‐population heterogeneity and the finite proliferative lifespan of these cells undermines reproducibility and limits the scalability required for consistent EV manufacturing. To mitigate these limitations, immortalisation and clonal expansion of the primary cells have been proposed as practical strategies to generate an infinite supply of uniform clonal cells, capable of supporting large‐scale and consistent EV manufacturing. However, even clonal cell lines can accumulate heterogeneity over time due to asymmetric cell division, epigenetic drift, or spontaneous mutations during expansion. Therefore, alongside rigorous selection of monoclonal cell lines with stable genomic and functional profiles, it is essential to maintain consistent passage numbers across EV production batches to minimise variability. Additionally, stringent testing to verify the identity and potency of the EV products remains critical to ensuring product quality and consistency.

Another major challenge lies in establishing robust potency CQAs. The molecular complexity of EV cargo makes elucidating the mechanisms of action inherently challenging. Establishing clear causal links between specific therapeutic effects and defined EV cargo components, or combinations thereof, requires rigorous mapping of EV attributes to biological outcomes. While engineered EVs overexpressing specific genes may offer a feasible strategy to simplify this mechanistic analysis, such manipulations also carry the risk of altering the broader EV molecular cargo in ways that remain insufficiently understood and require further clarification. Mechanistic studies are further complicated by the heterogeneous nature of OA itself, which encompasses post‐traumatic, metabolic, inflammatory and age‐related phenotypes [16]. Consequently, patient subgroups may respond differently to the same EV‐based therapy, underscoring the need to understand both cargo‐specific mechanisms and disease‐specific contexts to guide the development of targeted and effective EV therapeutics.

## 4 Hopes

Despite the challenges outlined, the trajectory of EV‐based therapies for OA is highly promising, driven by rapid advances in single‐vesicle analysis, multi‐omics, scalable manufacturing and artificial intelligence (AI). Together, these technologies provide a strong foundation for transforming EVs into predictable, scalable and clinically viable therapeutics.

Emerging single‐vesicle analysis technologies, such as microfluidics platforms, nano‐flow cytometry, super‐resolution microscopy and surface‐enhanced Raman scattering, allow high‐resolution profiling of individual EVs, revealing distinct subpopulations and their molecular signatures [17]. These approaches can characterise single‐molecule features including proteins, nucleic acids and lipids, providing unprecedented insight into EV heterogeneity and function. They are also informing the development of advanced EV tracing technologies.

Multi‐omics approaches, including genomics, transcriptomics, proteomics, lipidomics and metabolomics, offer detailed mechanistic insights to advance EV‐based therapies [18]. By capturing the comprehensive molecular landscapes of both EV cargo and OA pathology, these technologies enable the rational design of EV therapeutics, identification of robust potency markers and patient stratification based on predicted therapeutic responses. Such insights not only deepen mechanistic understanding but also pave the way for developing personalised EV therapies tailored to diverse OA phenotypes.

In parallel, substantial progress in manufacturing technologies is enhancing EV yield, purity and consistency [19]. Bioreactor systems have become increasingly sophisticated, offering precise control over culture conditions, supporting higher cell densities and producing significantly greater EV yield than traditional 2D static cultures. Strategies to boost EV production or enrich specific functional attributes are evolving rapidly and, as they become better characterised, are poised to be integrated into scalable, good manufacturing practice‐compatible platforms suitable for clinical and commercial manufacturing.

AI‐driven analytics are set to advance EV research and production by integrating complex datasets across single‐vesicle analysis, multi‐omics and manufacturing [17, 20]. In single‐vesicle studies, machine learning can classify EV subpopulations, reveal unique molecular signatures and detect rare vesicles. Applied to multi‐omics datasets, AI can uncover cargo‐function relationships, identify predictive biomarker‐outcome correlations and support patient stratification for personalised therapies. In manufacturing, AI enables real‐time monitoring, optimises cell culture and EV isolation parameters, reduces batch‐to‐batch variability and facilitates automated, data‐driven validation of EV purity, identity and functional potency.

Collectively, these advances offer a roadmap towards the development of precise, effective and scalable EV‐based therapies for OA, with the potential to transform both mechanistic understanding and clinical management of the disease. With a globally coordinated effort, there is hope that EVs may become a clinical reality in OA treatment in the next 5–10 years as a standalone or complementary therapy.

## Conflicts of Interest

The authors declare no conflicts of interest.

## Data Availability

Data sharing not applicable to this article as no datasets were generated or analysed during the current study.
